# The study of potable water treatment process in Algeria (boudouaou station) -by the application of life cycle assessment (LCA)

**DOI:** 10.1186/2052-336X-11-37

**Published:** 2013-12-19

**Authors:** Messaoud-Boureghda Mohamed-Zine, Aksas Hamouche, Louhab Krim

**Affiliations:** 1 Environmental Genius Departement, Engineering Sciences Faculty,Boumerdes University, Boumerdes, Algeria

**Keywords:** Life cycle assessment (LCA), Water treatment process, Potable water, Simapro6

## Abstract

Environmental impact assessment will soon become a compulsory phase in future potable water production projects, in algeria, especially, when alternative treatment processes such sedimentation ,coagulation sand filtration and Desinfection are considered. An impact assessment tool is therefore developed for the environmental evaluation of potable water production. in our study The evaluation method used is the life cycle assessment (LCA) for the determination and evaluation of potential impact of a drink water station ,near algiers (SEAL-Boudouaou^a^).

LCA requires both the identification and quantification of materials and energy used in all stages of the product’s life, when the inventory information is acquired, it will then be interpreted into the form of potential impact “ eco-indicators 99” towards study areas covered by LCA, using the simapro6 soft ware for water treatment process is necessary to discover the weaknesses in the water treatment process in order for it to be further improved ensuring quality life. The main source shown that for the studied water treatment process, the highest environmental burdens are coagulant preparation (30% for all impacts), mineral resource and ozone layer depletion the repartition of the impacts among the different processes varies in comparison with the other impacts. Mineral resources are mainly consumed during alumine sulfate solution preparation; Ozone layer depletion originates mostly from tetrachloromethane emissions during alumine sulfate production. It should also be noted that, despite the small doses needed, ozone and active Carbone treatment generate significant impacts with a contribution of 10% for most of the impacts.

Moreover impacts of energy are used in producing pumps (20-25 GHC) for plant operation and the unitary processes (coagulation, sand filtration decantation) and the most important impacts are localized in the same equipment (40-75 GHC) and we can conclude that:

– Pre-treatment, pumping and EDR (EDR: 0.-6 0 kg CO2 eq. /produced m^3^) are the process-units with higher environmental impacts.

– Energy consumption is the main source of impacts on climate change.

– Chemicals consumption (e.g. coagulants, oxidants) are the principle cause of impacts on the ozone layer depletion.

– Conventional plants: pre-treatment has high GHG emissions due to chemicals consumption.

## Introduction

It is foreseen that, in the next decades, increased potable water consumption and freshwater resources depletion will cause a worldwide water scarcity problem [1] By 2025, nearly one third of the world population will suffer from a water stress situation (UNWW, 2003). Caring for the environmental is a necessity to ensure that the environment is properly managed in line with the rapid sustainable development of a nation.

In Algeria, water management poses a big problem for the authorities. The resources which are available are less than those which are required. The outdated fashion of water conveyance and insufficient storage capacity hinder the correct distribution of water to the consumers. The daily quota per inhabitant remains small in comparison with international norms. The collected water in 2000 is estimated at 6.074 billion m^3^,of which 3.938 billion for irrigation (65%), 1.335 billion for domestic use (22%) and 801 billion in industry (13%) The water management policy is not efficient. Sustainable development is closely linked with the environmental management where development satisfy the basic human necessity apart from sources such as forests, minerals, air quality, water quality and quantity, sufficient rainfall and stable environment temperature. The International Standardization Organization (ISO) had developed the standard of life cycle assessment (LCA), which is Life Cycle Assessment (LCA) is n the so-called ‘Code of Practice [2] life cycle impact assessment LCIA), and Life cycle interpretation.

The work presented in this paper aims at developing a tool based on the LCA approach which could be used systematically for the environmental evaluation of potable water production station of Boudouaou.

## Materials and methods

In our study we use the Simpro 6 software for the determination of environmental impact of products or services of the water potable treatment station.

SimaPro 6- is a program developed by the Dutch company PRé Consultants, which enables cycle assessment (LCA), using databases specific inventory (created by the user) its generic setup means use has expanded to analysis of processes and services.

1. Product design.

2. Development of key performance indicators.

3. Calculation of carbon footprints.

4. Environnemental Product declarations.

5. Environnemental reporting.

6. Determination of environmental impact of products or services.

### Method

**Figure 1:**
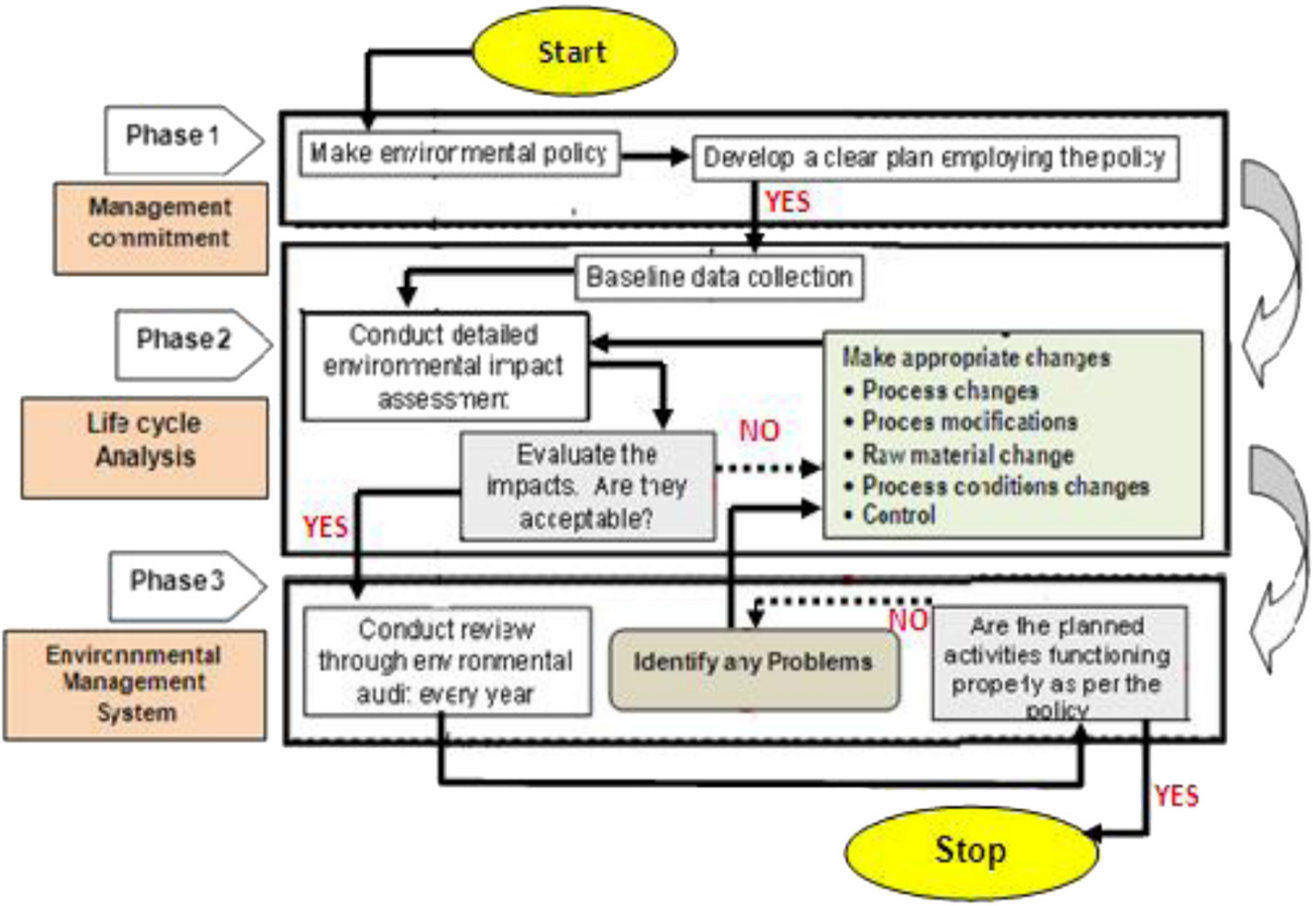
Environmental management system using LCA.

LCA can be used to analyze and compare several processes or systems through their contribution to global environmental impact [3] LCA is included in the ISO 14000 series and ISO 14040 series is a tool to gain environmental management decision support Environmental management would be effectively managed using LCA basically as summarized in Figure 1, Life Cycle Assessment (LCA) is a tool for the analysis of the environmental burdens of products or services at all stages of production, consumption, and end use (from “cradle to grave” [4-8].

LCA is a form of systems analysis for quantifying industrial process and products by enumerating flows of energy and material use, and wastes released to environmental burdens associated with energy and material use, wastes released to environment, and to evaluate alternatives for environmental improvements [9-12], a complete life cycle or ‘cradle to grave’ includes raw material extraction, processing, transportation, manufacturing, distribution, use, maintenance, recycling and final waste disposal [13,14].

While advances continue to be made, international and draft standards of the ISO 14000 series are, in general, accepted as providing a consensus framework for LCA and they consist of:

• International Standard ISO 14040) on principles and framework [15].

• International Standard ISO 14041 on goal and scope definition and inventory analysis [16].

• International Standard ISO 14042 on life cycle impact assessment [17].

• International Standard ISO 14043 on life cycle interpretation [18].

**Figure 2:**
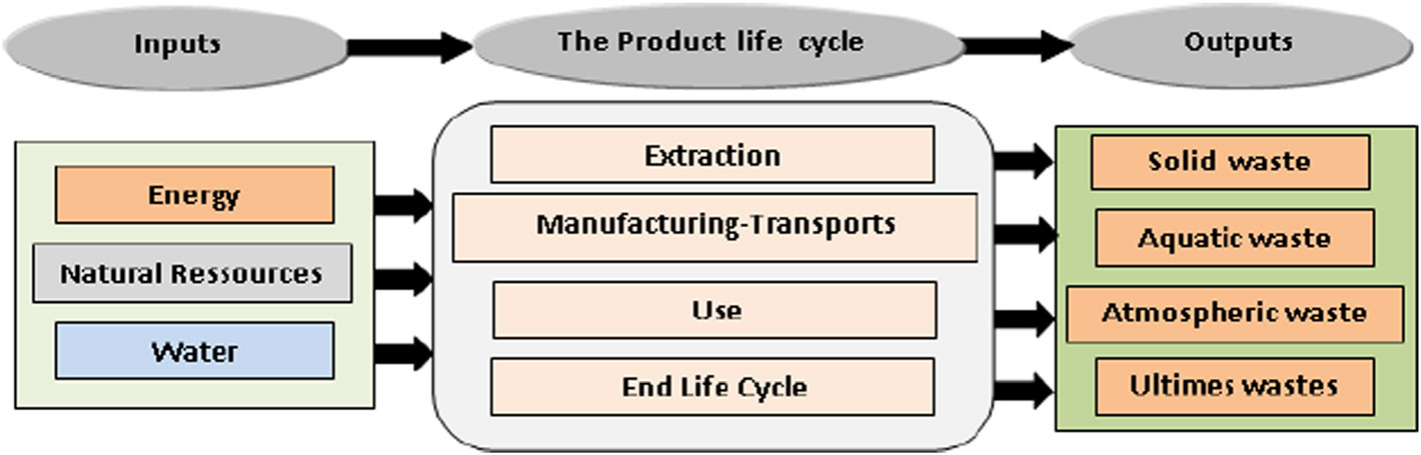
The products life phases.

The product life cycle in Figure 2 is shown in distinct phases, all of which interact with the environment. For most products, the period of use is far longer than the other periods, and there may also be periods of storage and non-use between the stages shown. Usually, but not always, these stages will be environmentally benign.

The main objectives of decision makers to initiate a LCA study are to [19-21].

▪ Provide a profile of the interactions of an activity (product, process or service) with the environment

▪ Contribute to the understanding of the overall and independent nature of the environmental consequences of human activities

▪ Provide decision makers with information, which quantifies the potential environmental impacts of activities and identifies opportunities for environmental improvements.

▪ Better decision support: LCA commonly is understood as a decision support tool. Backing the reported figures and results with uncertainty information allows assessing the stability of the result, and in some cases, a ranking order may be changed by considering the underlying uncertainty evidently, in a decision, information that changes a ranking of alternatives is of high importance, but also information on the stability of the result provided is immensely helpful.

▪ Transparency: The need to provide uncertainty informations on data and on LCA studies, bears the chance to clearly see the quality of data with that respect, and to identify, in a case study, “hot spots” in data quality.

▪ Quality competition: Transparency of data quality information entails a competition towards higher quality, less uncertain data. The transparently displayed uncertainty is especially if it is estimated to be too high, or if high uncertainty in the result does not allow giving a clear recommendation in the valuation, an incentive for reducing it and thus to improve the data quality. This “competition” aspect holds both within a case study and for independent data sources.

The high importance of LCA study of water treatment system is noticed as it would assist in preparing useful information for other processes directly or indirectly involving water This shows that it also have a substantial contribution to the environmental impact [22] to high population density areas in Algiers and Boumerdes cities and it is a potential project to be evaluated, using LCA.

In LCA studies, contributions by individuals to the environmental impact are also taken into consideration. The data show that need to double the volume of water used in agriculture to eradicate malnutrition in 2025 [23]. The fact remains that "the right to water is a palpable reality in the country, the daily amount of each individual being 60 liters to 165 liters a day as set by the United Nation [24].

Apart from that, Algeria is yet to have a LCA data inventory relating to water. LCA data inventory for water urgently needs to be compiled so that it can be used in the national level but the data can only be used as reference as the geographical differences of these researches is substantially diverse.

This study can help the head makers to understand Global and international environmental problems are still unsolved (dispersion of hazardous substances, increasing consumption of fossil fuels, increasing consumption of non-renewable mineral resources and over-exploitation of biological resources) [25,26] by conducting the LCA study in Algeria it will at least reduce these global problems at our level.

The objectives of this LCA research are:

▪ To gather comprehensive LCI of the processing system for the potable water.

▪ To conduct LCIA upon gathering all detailed LCI.

▪ To put forward a list of areas in the studied system for refurbishment purpose in order to reduce the stress on the environment.

This research is a case study for achieving the objective mentioned. Case study is concentrated on water treatment plant in (Algiers, Boumerdes) area only. High occupants density, agricultural and industrial areas are the main factors for selection for this case study. ISO 14040 series is used for this LCA research as this method is already consistent. Research methodologies are based on ISO documents and explanations about it in journals and books [27-30]. There are four phases (Figure 2) as stipulated in the international standard for conducting LCA [31] (***Goal and scope definition***,. The Functional unit of this study is defined as 1 liter of potable water.

▪ ***Life cycle inventory (LCI)****,* which quantifies the environmentally relevant inputs and outputs of the studied system, which is essentially a mass and energy balance of each unit, or smaller, process within the larger system. ISO has provided a general framework for the inventory analysis,This research uses the Simapro 6 software. This software contains European data. Basically, the primary data is collected directly from the manufacturers, the secondary data is obtained from the Simapro 6 software and tertiary data derived from calculation.

▪ ***Life cycle impact assessment (LCIA)*,** interpret the inventory results into their potential impacts on the areas of protection of the LCA.

▪ ***Life cycle assessment and interpretation (LCAI)*,** the last phase of an LCA and aim of this phase is to reduce the amount of data gathered during the LCA study to a number of key issues which will be usable in a decision making process.

### Water treatment process analysis

This research will be conducted on water treatment plant located in Boumerdes country. Raw water is extracted from the keddara made, its area is 93 Km^2^ and its Capacity is 145. 6 Billion per m^3^, with a treatment capacity of 450.000 m^3^/j , in the Table 1 we see the water quality before and after the treatment, and its easy to note that the potable water quality is conform to the world health organization (WHO).

**Table 1:** Water quality parameters before and after treatment

**Water quality parameters**	**Unit**	**Parameters before treatment**	**Parameters after treatment**
turbidity	NTU	3 - 3,5	0,2
Suspended matter	Mg/L	7,2	1,024
pH		8	7,96
Temperature	°C	13	12,5
Bacteria	UFC/1 Ml	505,	2
algae	(algae/Ml)	797	27
Hardness	^1^°F	40,6	40,2
Alkalinity°	°F	16	15,45
Organic matter	MgO/L	2,15	1,38
iron	Mg/L	0,11	<0,02
chlorophyll « a »	μg/l	1,2	0,5

The table informs us about the physic-chemical water parameters of “Keddara dam” before and after treatment, the results are indicators of the quality of the raw water (good quality mineral “low” and organic “very low”, the hardness is just acceptable and physical aspects are the sign of a good surface water quality.

^1^°F : French degre = 10mg/L of CaCO_3._

The Table 1 shows the principals physic-chemicals parameters of hard and treated water and we Note that the treatment process decreases, in accordance with global norms health, which originally turbidity is very low “5” NTU a great decrease, the suspended matter and the organics substances and bacteria, and une grande diminution of iron but the alkalinity and hardness water stay constant.

At the scale of the water treatment process, the tool focuses on each unitary treatment step of the process and tracks down the most penalizing technologies or products. This reveals the environmental weak points of the water treatment process and leads to the identification of sustainability improvement strategies.

### The water treatment process life cycle system case study

**Figure 3:**
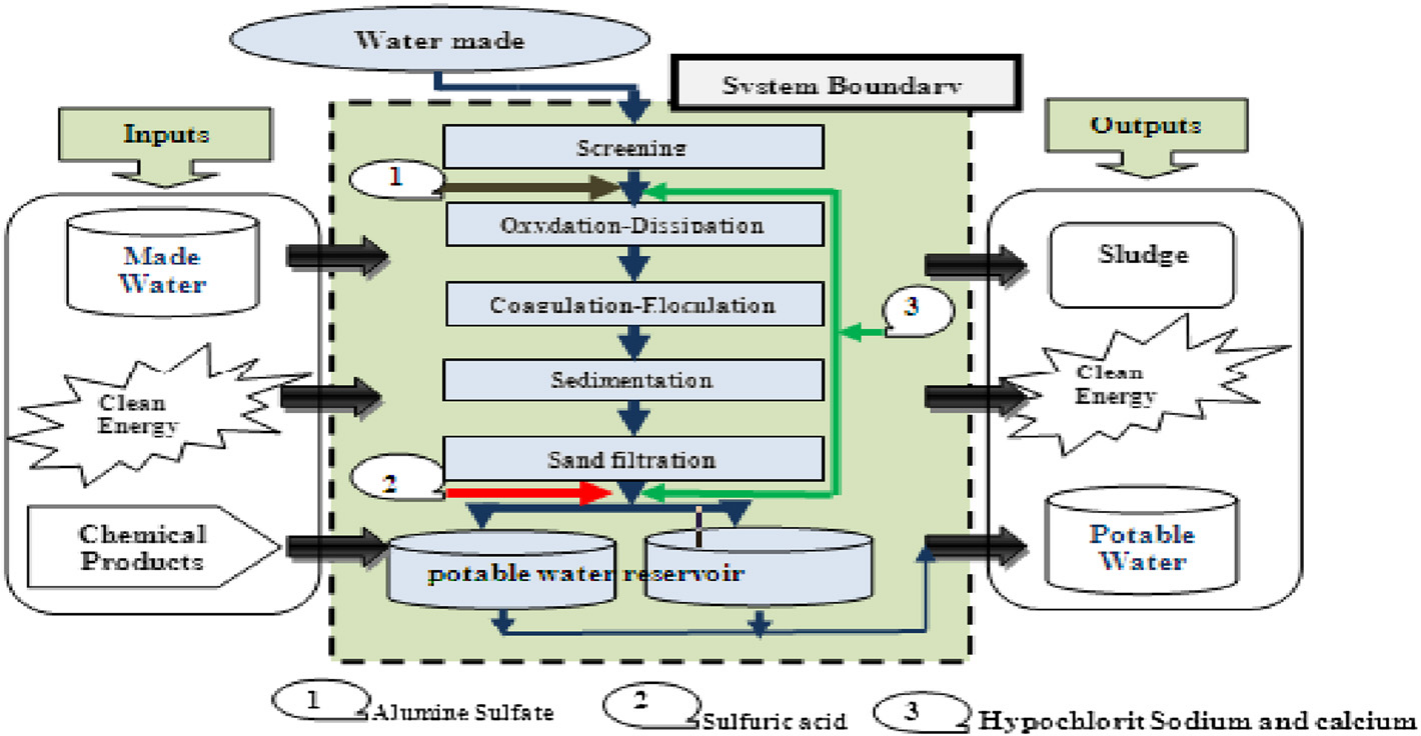
System boundary of production of potable water (Boumerdes-Algeria).

This LCA research will be conducted on water treatment plant located in Boumerdes country Raw water extracted from a water dam will go through the following process in the water treatment plant on Figure 3.

▪ ***Screening***, to remove floating big sized rubbish on the surface of the water. so the screener is near the dam it must protect the canalization until the treatment plant. but the screening is independent of the treatment plant, its management is supported with the dam.

▪ ***Water depression and oxidation of iron****.*

▪ ***Adsorption*** of micropolluants on carbon actived powder.

▪ ***Prédisinfection*** with chlorine.

▪ ***Coagulation and flocculation***, coagulation process is a process of forming particles called floc. Coagulant need to be added to form floc. The principal coagulants that is normally use include Aluminium Sulphate(70 g of Al_2_(SO_4_)_3_ per m^3^ Tiny flocs will in turn attract each other while at the same time pulling the dissolved organic material and particulate to combine, forming a big flocculant particle. This process is called flocculation.

▪ ***Sedimentation***, floc produced will settle on the base of the sedimentation basin (Pulsator decantor) with lamelles. The accumulation of flock settlement is called sludge.

▪ ***Filtration***, part of the flock which does not settle in the sedimentation basin will go through filtration. Water passing through filtration consisting of sand layers.

▪ ***Neutralization****:* add the sulfuric acid to neutralize the treated water Ph.

▪ ***Disinfection,*** process is needed to eliminate pathogen passing through the filters. Among the chemicals used for the disinfection are: sodium hypochlorite and calcium hypochlorite.

The input and output inventory of the life cycle system, including monthly consumption of land, water, energy and materials, as well as soil, water and air emissions, will derived from on-site investigations at water treatment plant. The studied water treatment process is dedicated to bacteria removal of surface water with high organic content and low hardness. (desinfection). The electricity required by the different treatment steps has been accounted considering the Sonelgaz (Algerian electric society) average production mix.

## Results and discussion

The decommissioning phase of potable water, production plant and the transport of raw, materials are negligible in comparison with the plant operation phase [31,32]. They are not accounted for in the LCA.

The steps responsible for most of the GHG emissions throughout the water treatment process life cycle are the chemicals products for coagulation and remineralization (soda, lime, sulfuric acid). The impacts of the successive treatment steps (coagulation, decantation, sand and filtration) are due to the production of the electricity required by these treatment steps. All together, the production of the electricity required by the complete treatment process is the second impact source after lime production. As stated in the literature review, the impact of the construction phase on climate change is low in comparison with the operation phase (5% of the total water treatment process impacts).

A “traditional” Green houses gases emissions assessment would have been limited to the plant itself, with Coagulation-flocculation appearing as the most penalizing step and off-site chemicals production are not been accounted for. Therefore, the LCA approach allows to quantify this so-called “pollution transfer” phenomenon and provides an unbiased and complete overlook of the water treatment impacts.

### Comparison between impact categories

**Figure 4:**
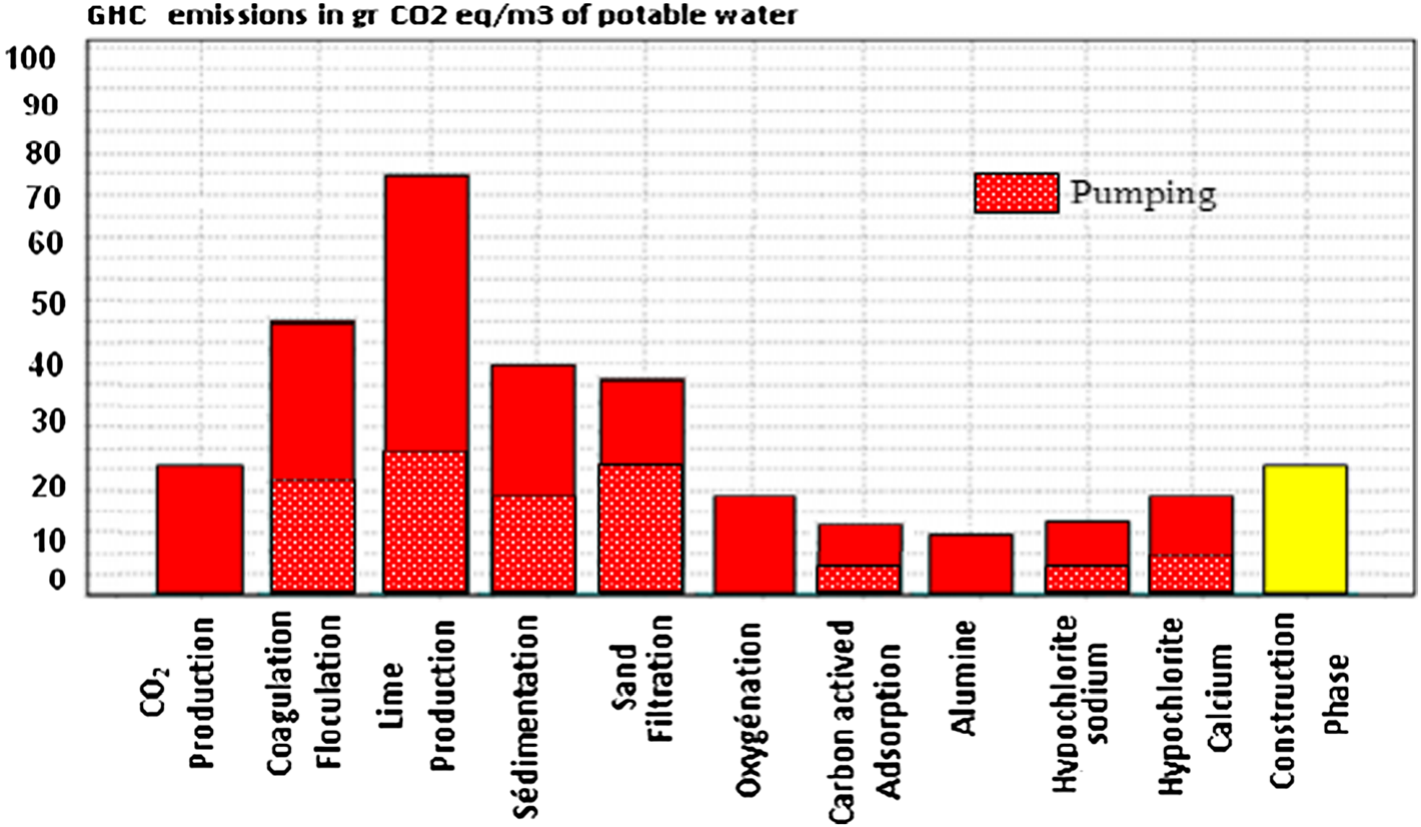
Global warming potential for each step of the potable water production process life cycle.

Besides a quantitative assessment, evaluating the relative contribution of each life cycle step to the impacts of the water treatment process is of prior importance. In Figure 4, the impact value of each treatment step is expressed in percent of the total water treatment process impact. The previous conclusions on global warming can now be reconsidered for the other impact categories.

**Figure 5:**
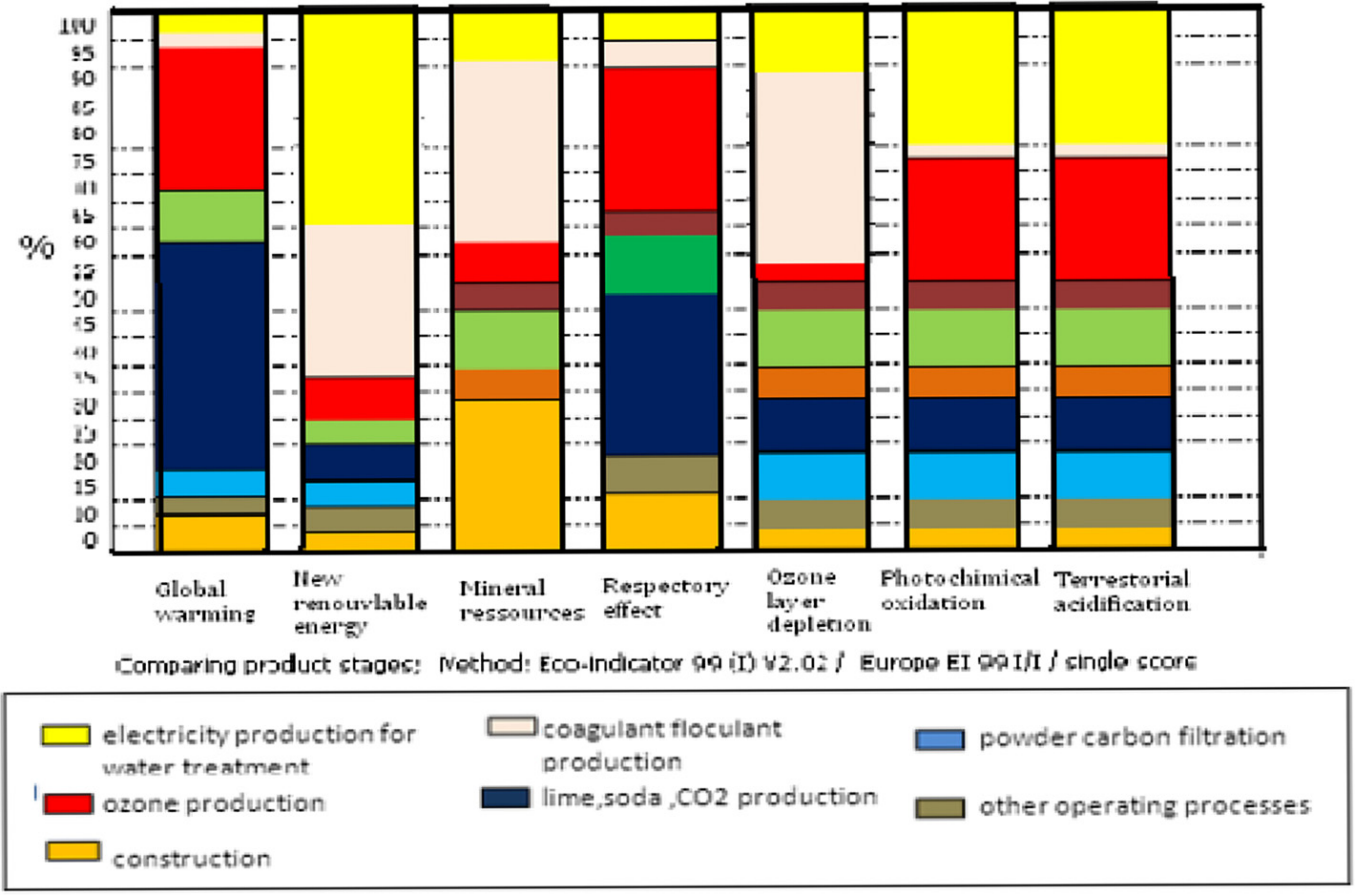
Contribution of the most penalizing steps to the impacts of the water treatment.

In The Figure 5 we show the impact caused by each operation of drinking water treatment in relation to eco-indicators 1999 The resulting scores provide an indication of areas for product improvements.

For the studied water treatment process, the steps carrying the highest environmental burdens are coagulant production preparation (more than 30% for all impacts), For mineral resource and ozone layer depletion the repartition of the impacts among the different processes varies in comparison with the other impacts. Mineral resources are mainly consumed during alumine sulfate solution preparation Ozone layer depletion originates mostly from tetrachloromethane emissions during alumine sulfate solution preparation. It should also be noted that, despite the small doses needed, ozone and active carbone treatment generate significant impacts with a contribution of 10% for most of the impacts.

**Figure 6:**
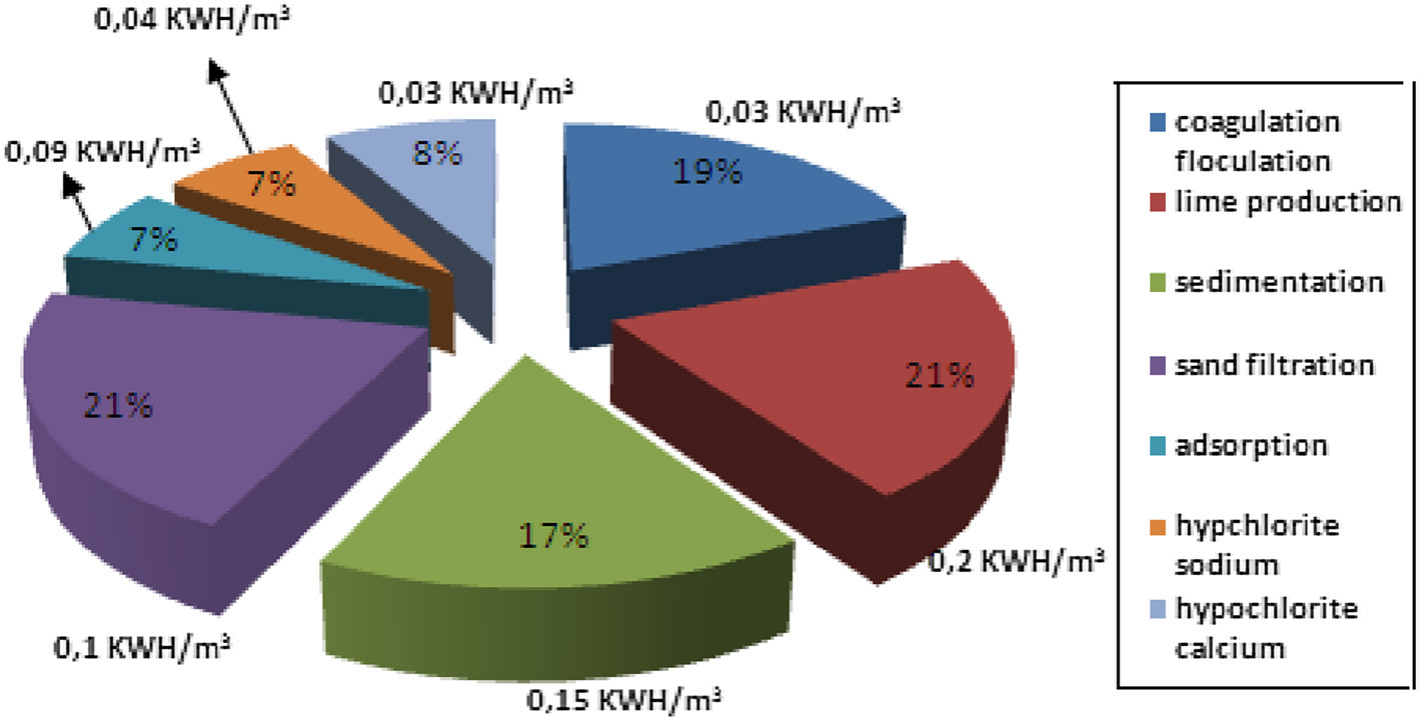
Proportion of pumping energy for each equipment of water treatment.

The electricity consumption, in Figure 6, of the water treatment process is mainly due to high speed decantation coagulation chemicals production for remineralization (20% for most of the impacts), filtration, and ozone production. Although it also consumes electricity, ozone production is evaluated separately because it corresponds to a complete chemical production process with multiple inputs (air, oxygen, heat, electricity) and outputs (gaseous emissions, residual ozone).

Large doses of coagulant, lime, sulfuric acid and soda are needed to reach potable water quality requirements.

Their production is responsible for more than 50% of impacts generated during the water treatment process life cycle. This is mainly due to the energy requirements of the chemicals production process and to gaseous emissions during chemicals production. As an example, lime production generates large amounts of GHG, and coagulant production leads to important ozone layer depletion impacts.

#### The average electricity consumption of specific pumping electricity used in the water treatment

The great proportion of the potential impact is due to the energy expended by the electricity consumed by pumps and the average electricity consumption in a wastewater treatment facility, h/m3 0.47 kilowatts, and Between 30 and 50% of specific pumping electricity used in the water distribution system, 0.5-0.7 kW h/m3 [33] in Figure 6 we find that Treated water pumping energy is a little close to 0.67 kWh/m3 while pumping electricity used in the specific water system, 0 0.5 to 0, 7 kW h/m3was found by Tarantini [34].

However, based on this study’s results, no correlation can be live, established operational impacts between and plant capacity. An example in this sense is Horgan plant [35], which despite is smallest water production, has higher energy use.

It is necessary to note that the most important aspects to emphasize, is that are similar to work realizes [36] are:

Pre-treatment, pumping and EDR (EDR: 0.-6 0 kg CO_2_ eq./produced m3 )are the process-units with higher environmental impacts.

Energy consumption is the main source of impacts on climate change.

Chemicals consumption (e.g., coagulants, oxidants) is the principle cause of impacts on the ozone layer depletion.

Conventional plants: pre-treatment has high GHG emissions due to chemicals consumption.

High extraction impacts due to pumping -Low chemicals and energy consumption in pre-treatment and GAC filtration.

75% the disinfection carbon footprint 5% the plant’s carbon footprint 40% the disinfection impacts on Ozone layer depletion 90% the plant’s impacts on ozone layer depletion.

### LCA case study limitation

There are a few limitations in this LCA case study. The following are the identified limitations:

▪ This research is concentrated on treatment plant in the Algiers- Boumerdes areas only. As such it does not depict accurately the environmental impact that might be produced by water treatment in the entire country.

▪ Confidentiality issues may arises that limits the data needed for the research. Transparency factor is a usual factor that happens in conducting LCA research [37,38].

▪ Data collection is the most time-consuming part in an LCA. This is due to the involvement of many plants and dealing with human behavior in order to convince them that the research is not to reveal the flaws of management but just as educational research.

▪ The degree of confidentiality and/or anonymity that will be provided. Include information on the extent to which and the manner in which records identifying the participant will be kept confidential, including any limits on confidentiality (for example, legal reporting requirements [research ethics boards, 2010].

▪ Average and/or typical data is used. These limitations together with the common data quality problem encountered in LCA studies contribute to the level of uncertainty of the results. The most appropriate ways to overcome some or all of these limitations need to be further investigated to enhance the use of LCA in assessment of water recycling options [39].

▪ The LCA results are dependent on t he geographic area from which the data. thus, the LCA conducted in Western country can not be exploited in an American country regardless of variations related to the geographical context (e.g., y dropower is the main source of energy in Quebec while Europe uses other sources of energy such as nuclear).

## Conclusion

This research is still in its development stage. Inventory data from water treatment process are still being collected from each plant involved. The main fact that wanted to be put forth by this paper is the advantage and significant of LCA research in the water treatment process in Algeria, the importance of water to human life is undeniable. Without water, human cannot continue their livelihood. Water drank by individual gives impact to the environment unknowingly to them [40]. Studies from [41-45] are the research on the water m8treatment process such as the treatment method (standard water treatment process such as those in practice in Algeria are proven research that water treatment process also contributes to the environmental impact).

This research has the merit of taking into account the impacts caused by the consumption of fresh water and try to quantify them using the same processes as those existing in the standard LCA models.

In response to stakeholders needs, a LCA tool has been developed for the environmental evaluation of potable water supply scenarios with various project conditions. This is made possible by modeling each unitary water treatment step as a function of the local context. Different water treatment processes, plants and potable water supply systems are then analyzed in order to present the main results.

Finally, The principal characteristic of supercial water of Boudouaou Dam (Algeria) is relatively the very low turbidity under “3,5 NTU” during nine months of the year, for removal this turbidity ,we need a small quantity of coagulant then we obtain the formation of a very small quantity of aluminum hydroxide and mud in the pulsator clarifier for reducting the potential impact of pumping we can, this led us to experiment the bypass of the decanter and to remove sludge and aluminum hydroxide in filter sand (particle size: 0.6 mm and porosity: 0.38), which had the positive effect of reducing the potential impact less than 60% the decanter at 60%.

## Endnote

^a^SEAL –Boudouaou : société des eaux d’Alger (water algiers society).

## Competing interests

The authors declare that they have no competing interests.

## Authors ‘contributions

All authors read and approved the final manuscript the second author manupuled the simapro logitiel and collects data inventory of impact factors the third author participates in the comments of the results and the recommendations.

## References

[B1] Shiklomanov IA Comprehensive assessment of the freshwater resources of the world: assessment of water 1997

[B2] Bonton A Bouchard CA Barbeau BB Jedrzejak S Comparative life cycle assessment of water treatment plants Desalination 2012 11 42 54

[B3] Jolliet O Saadé M Crettaz P Analyse du cycle de vie. Comprendre et realiser un ecobilan, Presses Polytechniques et Universitaires Romandes Int J Life Cycle Asses 2005 11 3 200 210 10.1065/lca2004.08.171

[B4] ROUSSEAUX P Analyse du Cycle de Vie (ACV), G 5500 Techniques de l’ingenieur 2005 11 noG5500, Note(s) G5500.1 G5500.4

[B5] Higgins AEC Life cycle assessment: a tool for environmental management 1996 Calgary, Alberta: Faculty of Environmental Design. The University of Calgary 121

[B6] Joshi S Comprehensive product life-cycle analysis using input output techniques 1999 pittsburg university,Pennsylvania: Thesis “doctor of pylosophy inpublic policy and management

[B7] Ross S Evans D Use of life cycle assessment in environmental management Environ Manage 2002 11 132 142 10.1007/s00267-001-0046-7 11740629

[B8] Almeida CMVB Rodrigues AJM SH B Giannetti BF Emergy as a tool for ecodesign: evaluating materials selection for beverage packages in brazil J Cleaner Prod 2010 11 32 43 10.1016/j.jclepro.2009.03.019

[B9] Mo W Embodied energy comparison of surface water and groundwater supply options Water Res 2011 11 17 5577 5586 Elsevier 10.1016/j.watres.2011.08.016 21889184

[B10] Khan F Raveender V Husain T Effective environmental management through life cycle assessment J Loss Prev Process Ind 2002 11 455 466 10.1016/S0950-4230(02)00051-7

[B11] Krozer J Vis JC How to get LCA in the right direction? J Cleaner Prod 1998 11 53 61 10.1016/S0959-6526(97)00051-6

[B12] Consoli A Guide Lines for Life-Cycle Assessment: A ‘Code of Practice 1993 Pensacola, FL, USA: Society of Environmental Toxicology and Chemistry SETAC

[B13] Klöpffer W The role of SETAC in the development of Life Cycle Assessment Int J Life Cycle Assess 2006 11 Supplement 1 116 122

[B14] ISO 14040 Environment Management-Life Cycle Assessment-Principles and Framework 2008 Geneva, Switzerland: International Standard Organisation

[B15] ISO 14041 Environment Management-Life Cycle Assessment, Goal and Scope Definition and Inventory Analysis 1998 Geneva, Switzerland: International Standard Organisation

[B16] ISO 14042 Environment Management-Life Cycle Assessment-Life Cycle Impact Assessment 2000 Geneva, Switzerland: International Standard Organisation

[B17] ISO 14043 Environment Management-Life Cycle Assessment-Life Cycle Interpretation 2000 Geneva, Switzerland: International Standard Organisation

[B18] Allen D Consoli F Davis G Fava J Warren J Public Policy Applications of Life Cycle Assessment 1997 Brussels: Society of Environmental Toxicology and Chemistry (SETAC), SETAC Press

[B19] Miettinen P Hamalainen RP How to benefit from decision analysis in environmental life cycle assessment Eur J Oper Res 1997 11 279 2 4 10.1016/S0377-2217(97)00109-4

[B20] Ya M-J Humphreys J Nicholas Holden M An evaluation of life cycle assessment of European milk production J Environ Manage 2011 11 3 372 379 10.1016/j.jenvman.2010.10.025 21055870

[B21] Schulz MA Streamlined sustainability assessment tool for improved decision making in the urban water industry Integr Environ Assess Manag 2011 DOI: 10.1002/ieam.247, http://onlinelibrary.wiley.com/10.1002/ieam.247 21751340

[B22] Mila I Canals L Chenoweth J Chapagain AK Orr S Antón A Clift R Assessing freshwater use impacts in Life Cycle Assessment: Part I—inventory modelling and characterisation factors for the main impact pathways Int J Life Cycle Asses 2009 11 1 28 42 10.1007/s11367-008-0030-z

[B23] Rijsberman FR Water scarcity: Fact or fiction? Agric Water Manage 2006 11 1-3 5 22 10.1016/j.agwat.2005.07.001

[B24] Hermanowicz SW Sustainability in Water Resources Management: Changes in Meaning and Perception Sustainability in Water Resources 2008 eScholarship University of Columbia 279 303 Volume 3 http://escholarship.org/uc/item/9h48p02k

[B25] Hauschild M Why Life Cycle assessment? Department of Manufacturing Engineering and Management, Denmark Technical University of Denmark Int J Life Cycle Asses 2007 11 1 24 33 10.1065/lca2006.12.287

[B26] Loiseau E Environmental Impacts Evaluations Methods of Water Use Equipe ELSA ((Environmental Lifecycle and Sustainability Assessment) 2010 Centre de Montpellier: AgroParisTech–ENGREF

[B27] Baumann H Tillman AM The Hitch Hiker's Guide to LCA: An orientation in LCA methodology and application 2004 Lund: Student litterature

[B28] Guinee JB Handbook of Life Cycle Assessment: Operation Guide to ISO Standards 2002 DORDRECHT: Kluwer Academic Publishers

[B29] Rebitzer G Ekvall R Frischknecht D Hunkler G Norris T Rydberg W-P Schmidt S Al *Life Cycle Assessment Part* 1: Framework, goal and scope definition, inventory analysis, and applications Environ Int 2004 11 701 720 10.1016/j.envint.2003.11.005 15051246

[B30] LCA](ISO ,14040) Assessing freshwater use impacts in Life Cycle Assessment: Part I—inventory modelling and characterisation factors for the main impact pathways Int J Life Cycle Asses 2009 11 1 28 42 http://dx.doi.org/10.1007/511367-008-0030-2 Outdoor Industry 10.1007/s11367-008-0030-z

[B31] Friedrich E Environmental Life Cycle Assessment of potable water production, Master thesis of Chemical Engineering 2001 South Africa: University of Natal

[B32] Raluy RG Serra L Uche J Valero A Life Cycle Assessment of water production technologies of different commercial technologies (MSF, MED, RO) Int J Life Cycle Asses 2005 11 5 346 354 10.1065/lca2004.09.179.2

[B33] Sahely HR Dudding S Kennedy CA Estimating the urban metabolism of Canadian cities: Greater Toronto Area case study Can J Civ Eng 2003 11 468 483

[B34] Tarantini M Ferri F Life Cycle Assessment of drinking and wastewater treatment systems of Bologna City: Final results Proc. 4th InterRegional Conf. on Environmental Water 2000 Fortaleza, Brazil: International Commission on Irrigation and Drainage” ICID”

[B35] Racoviceanu AI Karney BW Kennedy CA Colombo AF Life-cycle energy use and greenhouse gas emissions inventory for water treatment systems J Infrastructure Syst 2007 11 4 261 270 http://dx.doi.org/10.1061/(ASCE)1076-0342(2007)13:4(261) 10.1061/(ASCE)1076-0342(2007)13:4(261)

[B36] Russell A Ekvall T Baumann H Life cycle assessment - introduction and overview J Cleaner Prod 2005 11 1207 1210 10.1016/j.jclepro.2005.05.008

[B37] Messaoud-Boureghda MZ Louhab K Study of the Environmental Impacts of Urban Wastewater Recycling (Case of Boumerdes -Algeria by the Life Cycle Assessment Method Asian J Chem 2012 11 1 339 344

[B38] Louise C Annette D Gill B **Confidentiality and informed consent: Issues for consideration in the reservation of and provision of access to qualitative data archives** Forum Qual Soc Res 2000 11 3 7

[B39] Marín D CET aqua (Water technology Centre) Life Cycle Assessment and Water Issues International Summer School for the Environment 2012 Girona: Universitat de Girona

[B40] Poch M Introduction to water cycle Life Cycle Assessment and Water Issues 2012 Italia: XII international summer school for the environment Universitat de Girona

[B41] Friedrich E Environmental life cycle assessment of potable water production Water Sci Tech 2002 11 9 29 12448449

[B42] Landu L Environmental life cycle assessment of water use in South Africa: The Rosslyn industrial area as a case study Water SA 2006 11 2 249 256

[B43] Raluy R Serra L Uche J Life Cycle Assessment of Water Production Technologies - Part1: Life Cycle Assessment of Different Commercial Desalination Technologies (MSF, MED, RO) Int J Life Cycle Asses2005a 11 285 293 10.1065/lca2004.09.179.1

[B44] Raluy R Serra L Uche J Valero A Life Cycle Assessment of Water Production Technologies, Part 2: Reverse Osmosis Desalination versus the Ebro River Water Transfer Int J Life Cycle Asses2005b 11 346 354 10.1065/lca2004.09.179.2

[B45] Tarantini M Federica F LCA of drinking and wastewater treatment systems of Bologna city: Final results4th IRCEW Conference, Inter-Regional Conference on Environment-Water Fortaleza, Brazil 2001 URL: http://www.funarbe.org.br/ircew/

